# Nitrofurantoin as an Add-On to Conventional Prophylaxis for the Treatment of Urinary Tract Infections in Kidney Recipients: A Prospective Cohort Study

**DOI:** 10.3390/jcm13175218

**Published:** 2024-09-03

**Authors:** J. Ahuixotl Gutiérrez-Aceves, Felipe Alexis Avalos-Salgado, Jorge Ivan Gamez-Nava, Laura Gonzalez-Lopez, Sergio Antonio González-Vázquez, Reynaldo Arellano-Cervantes, Mario Alberto Mireles-Ramírez, Jazmin Marquez-Pedroza, Melissa Ramirez-Villafaña, Eli Efrain Gomez-Ramirez, Fabiola Gonzalez-Ponce, Ana Miriam Saldaña-Cruz, Norma Alejandra Rodriguez-Jimenez, Ernesto German Cardona-Muñoz, Sylvia Totsuka-Sutto, Juan Manuel Ponce-Guarneros

**Affiliations:** 1 Programa de Doctorado en Farmacología, Centro Universitario de Ciencias de la Salud, Universidad de Guadalajara, Guadalajara 44340, Mexico; 2 Research Group for Factors Related to Therapeutic Outcomes in Autoimmune Diseases, Centro Universitario de Ciencias de la Salud, Universidad de Guadalajara, Guadalajara 44340, Mexico; 3 Hospital de Especialidades CMNO, Division de Investigación en Salud. Av. Belisario Domínguez 999, Independencia Oriente, Guadalajara 44340, Mexico; 4 Programa de Maestria Salud Publica, Departamento de Salud Pública, Centro Universitario de Ciencias de la Salud, Universidad de Guadalajara, Guadalajara 44340, Mexico; 5 Instituto de Terapéutica Experimental y Clínica, Departamento de Fisiología, Centro Universitario de Ciencias de la Salud, Universidad de Guadalajara, Guadalajara 44340, Mexico; 6 Hospital General Regional 110, Instituto Mexicano del Seguro Social, Guadalajara 44716, Mexico; 7 Neurosciences Division, Western Biomedical Research Center, Mexican Institute of Social Security, Guadalajara 44340, Mexico; 8 Instituto Mexicano del Seguro Social, Unidad de Medicina Familiar No. 97, Magdalena 46474, Mexico

**Keywords:** kidney transplant, nitrofurantoin, urinary tract infection, antimicrobial resistance, prophylactic treatment, cohort study

## Abstract

Urinary tract infections (UTIs) constitute one of the main complications in kidney recipients, increasing both morbidity and mortality. Due to the resurgence of antimicrobial resistance, new prophylactic approaches are being investigated. Nitrofurantoin is an antibiotic from the nitrofuran group that is effective against several Gram-negative and Gram-positive organisms; hence, there has been a resurgence in its prescription for treating MDR pathogens. **Objectives**: This study aims to assess the effectiveness of nitrofurantoin as an add-on to conventional therapy (amikacin + ceftriaxone or cefotaxime) for the treatment of urinary tract infections in kidney recipients. **Methods**: In a prospective cohort study, we included patients who received a kidney in a tertiary-care hospital. According to the intensive care specialist, group 1 patients were treated with the conventional prophylactic treatment plus nitrofurantoin as an add-on. Group 2 patients were treated only with the conventional prophylactic treatment. They were followed-up for 3 months, and the incidence of urinary tract infections was reported. **Results**: The UTI incidence for group 1 at 3 months was 20.6%, and for group 2, it was 20.0%; no statistical difference between treatments was observed (*p* = 0.9). The most commonly isolated pathogens were *E. coli* (28.5) and *K. pneumonie* (28.5%). The factor most associated with developing a UTI was female gender (aHR: 7.0; 95% IC 2.3–20.9, *p* < 0.001). **Conclusions**: In our cohort study, nitrofurantoin as an add-on in conventional therapy did not prove to be effective in preventing UTI development; therefore, other treatment options should be considered as a part of prophylactic treatment.

## 1. Introduction

Urinary tract infections (UTIs) constitute one of the main complications in kidney recipients, increasing both morbidity and mortality [1,2]. The incidence of UTIs post kidney transplant has been estimated at 25% to 75% of patients [3,4,5]. 

Gram-negative bacteria are the most frequent causal agents of UTIs in kidney recipients, with *Escherichia coli (E. coli)* as the most frequent pathogen isolated [6]; other commonly isolated bacteria are *Pseudomonas aeuroginosa* (*P. aeuroginosa*), *Enterobacter cloacae* (*E. cloacae*), and *Klebsiella* species (*Klebsiella* spp.) [7]. However, Gram-positive infections such as *Enterococcus* spp. can also be found as causal agents [8]. UTIs can lead to complications, such as bacteremia, acute immune reactions resulting in impaired function or loss of allograft, and an almost double length of time of hospitalization [9,10,11]. The risk factors associated with kidney recipients developing a UTI are an increased susceptibility to infections due to immunosuppression secondary to treatments [8], abnormalities of the urinary tract, some comorbidities such as diabetes mellitus or hypertension [12], female gender, advanced age, a second kidney transplant, renal calculi, and cadaveric donor kidneys [6,13].

Prevention of UTIs is required to avoid complications; prophylaxis lowers the risk of developing bacteriuria by 60% in patients who undergo a kidney transplant [14]. However, treatment regimens are very heterogeneous and are mostly based on the physician’s experience [15]. Approximately one-third (37%) of UTIs are caused by multidrug-resistant bacteria (MDR), making it a challenge to treat transplant patients [16,17]. Therefore, the effectiveness of other drugs used as prophylaxes must be investigated. Nitrofurantoin is an antibiotic from the nitrofuran group that acts by blocking protein synthesis in the ribosome, breaking DNA chains, and blocking the activity of acetyl-coenzyme A; it is commonly used to treat uncomplicated lower urinary tract infections, taken at doses of 100 mg orally twice daily [18]. It is effective against several Gram-negative and Gram-positive organisms, reporting clinical cure rates between 79 and 92% alongside high microbiological eradication rates of 80% to 92% in the general population; hence, there has been a resurgence in its prescription for treating MDR pathogens [18,19]. However, studies focusing on the use nitrofurantoin as a prophylactic agent in kidney recipients are scarce [20]. The objective of this cohort study was to evaluate the effectiveness of nitrofurantoin as an add-on to conventional therapy for the treatment of urinary tract infections post kidney transplants. 

## 2. Materials and Methods

### 2.1. Design of the Study and Clinical Setting

This study was a prospective cohort, where 70 patients who underwent renal transplant surgery from the post-surgical transplant care unit in a tertiary-care hospital were followed from admission to discharge and then up to 3 months after surgery. This cohort was assessed from December 2022 to December 2023.

### 2.2. Eligibility Criteria

We included patients who were in the Transplant Post-Surgical Care Unit (PCU) with immediate diagnosis of kidney transplant. These patients were ≥18 years old at the moment of the kidney transplant and presented a negative urine culture upon admission to the post-surgical transplant care unit; all the kidneys came from a living donor, either related or non-related. Patients were excluded if they had a ureteral stent placed during surgery, if their kidney came from a cadaveric donor, or if they presented any of the following post-surgical complications in the first 8 h: peri-graft hematoma, urinary leak, massive bleeding, and/or anuria. Patients were also excluded from this study if they presented with the loss of the kidney graft during their stay in the PCU or if, during their prophylaxis treatment, they showed moderate-to-severe side effects. Lastly, any patient who expressed their wish to stop participating in the study was eliminated from the cohort. 

### 2.3. Ethics

The approval of this cohort study was granted by the Ethics in Research Committee at the tertiary-care hospital, with the approval code of: R-2022-1301-159. This research protocol followed the Ethical Principles for Medical Research Involving Human Subjects described in the Declaration of Helsinki [21]. All patients willing to participate signed informed consent for participation in this study.

### 2.4. Study Development

In this study, we assessed and compared the rate of UTIs in 70 patients who underwent kidney transplants. Conventional prophylaxis, according to the hospital attention protocol, was 500 mg intravesical amikacin prior to surgery, which was administered to all patients, plus a third-generation cephalosporin (cefotaxime 1 g IV or ceftriaxone 1 g IV, two doses each) prior to surgery. In 3 cases of allergy to penicillin, these patients were prophylactically treated with a single dose prior to transplant of quinolone: levofloxacin 500 mg IV, one dose.

Two groups were formed: Group 1 patients received conventional prophylaxis treatment plus nitrofurantoin as an add-on. Nitrofurantoin was indicated by the attending physician after the kidney transplant, and it was administered orally at doses of 100 mg twice per day for a period of seven days after the kidney transplant. Group 2 patients solely received conventional prophylaxis treatment as described above. 

#### 2.4.1. Clinical Assessments and Follow-Up

Epidemiological and clinical data were ascertained at the following time points: PCU admission, PCU discharge, and 7 days, 2 weeks, 3 weeks, 1 month, 2 months, and 3 months after discharge. The collected information was as follows:

(a) Sociodemographic variables: gender, age, BMI, length of chronic kidney disease (CKD) diagnosis, etiology, type of donor, and the use of a substitutive donor;
(b) Chronic diseases: hypertension, diabetes mellitus type 2, obesity, etc.;
(c) Prophylactic treatment: antibiotics used;
(d) Safety: any adverse event that led to the suspension of any antibiotic used as prophylactic treatment;
(e) Clinical data: hematic biometry, blood chemistry, general urine examination, electrolytes, liver profile, and urine culture;
(f) Infection-related: causal pathogen, time passed between transplant and infection, and presence of resistance and/or susceptibility to antimicrobials indicated by antibiogram testing.

#### 2.4.2. Outcomes Measures

The main outcome in this cohort was the effectiveness of the prophylactic agents, both conventional and conventional + nitrofurantoin as add-on, which was assessed as the rate of UTIs at each visit. Pathogens from patients who reported a UTI were isolated, and if any microorganism showed resistance to 3 or more families of antibiotics, it was considered multidrug-resistant [22]. Secondary outcomes were the incidence of complications due to the surgery, alongside impaired function or loss of the allograft. The severity of the UTI was graded on a scale from 1 to 6, depending on the clinical symptoms [23].

### 2.5. Statistical Analysis

The urinary tract bacterial infections’ incidence rate was computed, each bacterial strain was identified, and their susceptibility/resistance was reported as the frequency and percentage. Independent Student’s *t*-tests were used for comparisons of the quantitative variables between groups (nitrofurantoin as add-on vs. solely conventional antibiotic prophylaxis). Chi-square tests (or Fischer exact tests if required) were used for the comparisons of the proportions between groups. The rate of UTIs was analyzed using the Kaplan–Meier method. Univariate and multivariate Cox proportional hazards regression models were used to assess potential predictors for urinary tract bacterial infections. The significance level was set at *p* ≤ 0.05. The analyses were performed using the statistical software SPPS Statistics Version 24.

## 3. Results

Table 1 compares the patients’ sociodemographic characteristics at baseline; the male sex was more prominent (75.7%), with a mean age of 34.1 ± 10.1. Almost all the patients had at least one comorbidity, with the most frequent being arterial hypertension (90.0%). The mean time since chronic kidney disease (CKD) diagnosis was 6.5 ± 5.0 years, and the most frequent etiology was unknown (78.6%). Almost all of them were receiving renal replacement therapy (92.9%). There were no significant differences between groups, except for the timing of the CKD diagnosis. 

We observed a total of 12 complications: 2 cases of hematomas; 2 cases of urinary leak; 4 cases of acute renal failure; 1 case of heart failure; 1 case of retarded graft function; 1 case of uncontrolled hypertension; and 1 case of pancytopenia. None of the patients rejected the allograft or died during this study.

### Incidence of Urinary Tract Infections

There were 14 cases of UTI observed during the cohort: 7 cases (19.4%) were reported in the nitrofurantoin group, and 7 cases (20.6%) were reported in the conventional treatment group (*p* = 0.9). The severity of all the UTIs observed in this study was grade 1. No significant difference was observed between groups.

**Figure 1:**
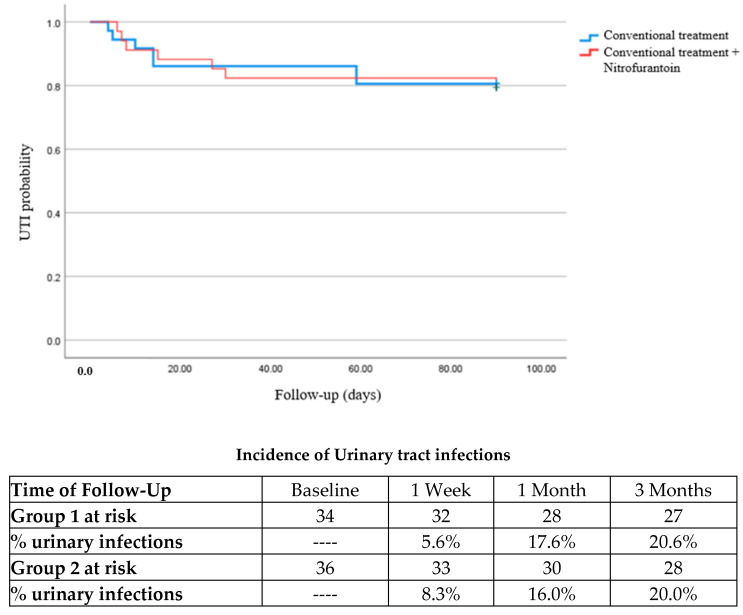
Comparison of UTI incidence by treatment.

Figure 1 shows the Kaplan–Meyer curve comparison of urinary infections in group 1 (*n* = 7) vs. group 2 (*n* = 7), where no significance difference was observed between them at 3 months (*p* = 0.9). In addition, we compared the incidence of urinary tract infections at different times: at 1 week post-transplant, group 1 presented two cases, and group 2 also presented two cases; hence, no difference was observed (*p* = 0.9). At 1 month after surgery, group 1 presented five cases, and group 2 presented six cases, although without a significant difference (*p* = 0.7).

**Figure 2:**
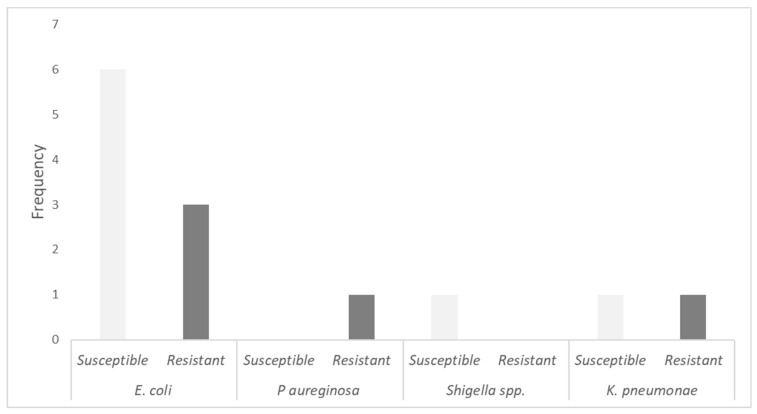
Etiology of urinary tract infections according to antibiotic susceptibility.

Table 2 lists the bacteria identified in our cohort; in group 1, there were seven UTI cases, and the most common pathogens isolated were *E. coli* (28.5%) and *K. pneumonie* (28.5%), followed by *E. coli* (ESBLs) (14.2%) and *Shigella* spp. (14.2%). Group 2 also reported seven UTI cases; most of these were due to *E. coli* (42.8%) and *E. coli* (ESBLs) (42.8%). The resistances and susceptibilities are also reported. Regarding multidrug resistance, 6/14 cases (42.9%) were due to MDR bacteria, distributed equally in both groups, 3/7 cases (42.9%) in group 1 and 3/7 cases (42.9%) in group 2 (Figure 2).

Table 3 shows a comparison between the patients who did present a urinary infection and those who did not develop an infection during follow-up. A higher proportion of female patients developed an infection (64.3% vs. 14.3%, *p* ≤ 0.001); however, no other variable showed a significant difference between patients, especially in the use of nitrofurantoin.

The results of the multivariate Cox risk analysis are shown in Table 4. In the model, a time-dependent variable was defined: the development of urinary tract infections. The covariables (potential confounders) selected to be tested in the unadjusted model (Enter Method) were female sex, BMI, comorbidities, years of CKD, and use of nitrofurantoin. The risk model showed a significant relation between urinary tract infection and female sex (HR = 8.7; 95% CI: 2.5, 29.8, *p* ≤ 0.001). However, no statistical associations were found with the other confounders. After adjusting these potential confounders using the stepwise method, only one variable remained significantly associated with urinary infections: female sex (aHR = 7.0, 95% CI: 2.3, 20.9, *p* < 0.001). Other risk factors associated with UTIs in patients who underwent renal transplant were multidrug-resistant bacteria; although it is not a host risk factor, it should be considered to optimize treatment.

## 4. Discussion

In this study, we compared the urinary tract infection incidence in patients who underwent renal transplant using nitrofurantoin as an add-on to conventional antimicrobial therapy (group 1) vs. patients using solely conventional antimicrobial therapy (group 2). We observed a total of 14 UTIs with an incidence of 5.7% after one week, 15.7% after 1 month, and 20% after 3 months. The most commonly isolated pathogen was *E. coli*.

### 4.1. Use of Nitrofurantoin

Due to the surge in antimicrobial resistance in bacteria, nitrofurantoin has been analyzed as a therapeutic option because of its low prevalence of bacterial resistance [15,18,24,25]. In our study, nitrofurantoin did not show any additional prophylactic effect on urinary tract infections compared with conventional treatment. In the nitrofurantoin group, we observed seven UTI cases, with an incidence of 5.6% after one week, 17.6% after 1 month, and 20.6% after 3 months; group 2 also showed seven cases of UTI, with an incidence of 8.3% after one week, 16.0% after 1 month, and 20.0% after 3 months. In another study, Memikoğlu K et al. analyzed the medical records of 156 patients who underwent renal transplantation, of which 34 patients received nitrofurantoin. Of those patients, 12/34 presented with a urinary tract infection, from which they concluded that it was ineffective as a prophylactic agent [20]. Halskov A et al. performed a retrospective cohort analyzing 571 renal transplants in order to understand the risk factors associated with urinary tract infections. Nitrofurantoin was prescribed in 7.7% of these records; however, none of the specific antibiotics were a protective factor for developing urinary tract infections [26]. Coussement J et al. analyzed the benefit of using antibiotics in kidney transplant recipients with asymptomatic bacteria with a follow-up of 1 year; here, only 5% of the antibiotic treatments used nitrofurantoin, and no difference was observed between using therapeutic antimicrobials and not at 1 year [27]. 

### 4.2. Urinary Tract Infections

In our cohort, we reported 14 cases of UTIs, with 7 cases in each group. The most common pathogen observed was *E. coli* (nine cases), from which four presented extended-spectrum beta-lactamases (ESBLs). It must be noted that those with *E. coli* who presented ESBLs were susceptible to nitrofurantoin, while those who were ESBLs-negative were resistant to the antibiotic. After *E. coli*, the second most frequent bacterium was *K. pneumonie,* which was resistant to nitrofurantoin. Lastly, one case of *P. aeruginosa* was reported, which was also nitrofurantoin-resistant; there was also an infection caused by *Shigella* spp., which was susceptible to nitrofurantoin. Coussement J et al. also reported *E. coli* as the main causal pathogen of UTIs in their cohort, followed by *Klebsiella* spp. [27]. The same pattern was observed by Halskov A et al., where *E. coli* was also the most prevalent causal agent followed by *K. pneumonie* [26]. The same results regarding the causal pathogen can be observed in other studies [15,20,25].

### 4.3. Risk Factors Associated with Urinary Infections

We assessed the role of nitrofurantoin use through bivariate and multivariate analysis, without observing any statistical difference. The only risk factor we observed in the multivariate analysis using Cox regression was being female, which increased the risk of developing a UTI sevenfold. Another risk factor associated with UTIs in patients who underwent renal transplant is multidrug-resistant bacteria [25]; in our study, 13/14 infections were due to multidrug-resistant pathogens. Another risk factor is worse graft function [15]; however, in our study, we did not assess the graft function. 

### 4.4. Strengths 

Our cohort focused on the prescription of nitrofurantoin as a prophylactic agent to treat UTIs in renal transplant patients; many other studies tend to overlook the specific use of nitrofurantoin and instead analyze the use of many antibiotics. Therefore, the proportion of patients using this antimicrobial is low and not enough to generate reliable evidence. We compared the use of nitrofurantoin with conventional treatment with a follow-up time of 3 months to analyze its effectiveness in treating nosocomial infections and late-onset infections. Additionally, in our cohort, all kidney recipients came from living donors, which marks a difference from other studies where cadaveric donors are considered, although they present a risk for developing a UTI.

### 4.5. Limitations

This study was conducted at a single center; therefore, the results cannot be wholly generalizable. In addition, microbiology and antimicrobial resistance patterns may also differ. Another limitation was the reduced sample size for each study group, reducing the statistical power of some analyses.

## 5. Conclusions

In this study, nitrofurantoin proved to be ineffective as a prophylactic agent for urinary tract infections in patients who underwent renal transplant. More studies are needed to assess nitrofurantoin’s effectiveness as a prophylactic option in urinary tract infections. Health professionals should consider assessing other prophylactic agents for treating post-transplant patients.

## Figures and Tables

**Table 1:** Sociodemographic and epidemiological characteristics.

Variables, *n* (%)	Total *n* = 70 (%)	Group 1 Nitrofurantoin as Add-on + C.P. *n* = 34 (%)	Group 2 Conventional Prophylaxis *n* = 36 (%)	*p*
Male sex, *n* (%)	53 (75.7)	26 (72.2)	27 (79.4)	0.4
Age (yrs), mean ± SD	34.1 ± 10.1	33.0 ± 7.8	35.2 ± 12.0	0.3
Age group, *n* (%)				0.3
18–30 (yrs)	29 (41.4)	15 (41.7)	14 (41.2)	
31–59 (yrs)	39 (55.7)	21 (58.3)	18 (52.9)	
≥60 (yrs)	2 (2.9)	0 (0.0)	2 (5.9)	
BMI, mean ± SD	24.2 ± 3.8	24.4 ± 4.3	24.0 ± 3.2	0.6
Comorbidities, *n* (%)	65 (92.9)	33 (91.7)	32 (94.1)	0.6
Arterial hypertension, *n* (%)	63 (90.0)	32 (88.9)	32 (92.2)	0.7
Diabetes mellitus type 2, *n* (%)	5 (7.1)	3 (8.3)	2 (5.9)	0.6
CKD diagnosis (yrs), mean ± SD	6.5 ± 5.0	5.1 ± 4.2	8.0 ± 5.5	0.019
CKD etiology, *n* (%)				N.C.
Unknown	55 (78.6)	31 (86.1)	24 (70.6)	
DM	4 (5.7)	2 (5.6)	2 (5.9)	
SAH	1 (1.4)	0 (0.0)	1 (2.9)	
Polycystic disease	3 (4.3)	1 (2.8)	2 (5.9)	
Glomerulonephritis	5 (7.1)	0 (0.0)	5 (14.7)	
Other	2 (2.6)	2 (5.6)	0 (0.0)	
Renal replacement therapy, *n* (%)	65 (92.9)	34 (94.4)	31 (91.2)	0.6
Related living donor, *n* (%)	51 (72.9)	27 (75.0)	24 (70.6)	0.6
Non-related living donor, *n* (%)	19 (27.1)	7 (15.0)	12 (29.4)	
Antibiotic prophylaxis				
Intravesical Amikacin, *n* (%)	70 (100.0)	34 (100.0)	36 (100.0)	N.C.
Ceftriaxone IV, *n* (%)	60 (85.7)	28 (82.3)	32 (88.8)	0.4
Cefotaxime IV, *n* (%)	7 (10.0)	4 (11.7)	3 (8.3)	0.8
Levofloxacin IV, *n* (%)	3 (4.3)	1 (2.9)	2 (5.5)	0.9

Abbreviations: C.P.: conventional prophylaxis; BMI: body mass index; CKD: chronic kidney disease; DM: diabetes mellitus type 2; SAH: systemic arterial hypertension; N.C.: not calculated; IV: intravenous; yrs: years. Qualitative variables are represented as frequencies and percentages, with quantitative variables as means and standard deviation (SD).

**Table 2:** Pathogens isolated in patients using nitrofurantoin.

Organism	Num. of Cases, (%)	Days until Infection	Susceptibility	Resistance
Pathogens isolated in patients using Nitrofurantoin				
*E. coli*	2 (28.5)	6 and 8	Ampicillin/Sulbactam, Carbapenems, Amikacin, and Nitrofurantoin	Amoxicillin, Ampicillin, Cephalotin, Cefuroxime, Ceftriaxone, Cefepime, Gentamicin, Quinolones, and Trimethoprim/Sulfamethoxazole
*E. coli* (ESBLs)	1 (14.2)	27	Carbapenems, Quinolones, and Nitrofurantoin	Cephalosporines
*K. pneumonie*	2 (28.5)	7 and 90	Amikacin, Ertapenem, Meropenem, and Carbapenems	Amoxicillin, Ampicillin, Ampicillin/Sulbactam, Cephalosporins, Quinolones, Trimethoprim/Sulfamethoxazole, and Nitrofurantoin
*Shigella* spp.	1 (14.2)	15	Ertapenem, Meropenem, Amikacin, and Nitrofurantoin	Trimethoprim/Sulfamethoxazole and Quinolones
Multiple infections *	1 (14.2)	30	----	----
Pathogens isolated in patients using conventional treatment				
*E. coli*	3 (42.8)	14, 59, 59	Carbapenems, Cephalosporins, Quinolones, Cefotaxime, Ceftazidime, Ceftriaxone, Amikacin, Meropenem, Ertapenem, Ampicillin/Sulbactam, and Nitrofurantoin	Trimethoprim/Sulfamethoxazole, Quinolones, Ampicillin, Cephalotine, Cefuroxime, Ceftriaxome, Cefepime, and Gentamicin
*E. coli* (ESBLs)	3 (42.8)	4, 5, 14	Carbapenems, Ciprofloxacin, and Nitrofurantoin	Cephalosporines, Quinolones, and Trimethoprim/Sulfamethoxazole
*P. aeruginosa*	1 (14.2)	10	Ertapenem and Meropenem	Nitrofurantoin, Quinolones, and Cephalosporins

Abbreviations: ESBLs: extended-spectrum beta-lactamases. * Caused by *E. coli* and *P. aeruginosa.* Qualitative variables are represented as frequencies and percentages.

**Table 3:** Comparison of patients who developed a UTI.

Variables, *n* (%)	Without Urinary Tract Infection *n* = 56 (100.0)	With Urinary Tract Infection *n* = 14 (100.0)	*p*
Female sex, *n* (%)	8 (14.3)	9 (64.3)	<0.001
Age (yrs), mean ± SD	33.0 ± 8.8	38.4 ± 13.7	0.1
BMI, mean ± SD	24.5 ± 3.9	23.2 ± 3.0	0.2
CKD diagnosis (yrs), mean ± SD	6.4 ± 5.0	7.0 ± 5.4	0.6
Related donor, *n* (%)	41 (73.2)	10 (71.4)	0.8
Comorbidities, *n* (%)	52 (92.9)	13 (92.9)	1.0
Arterial hypertension, *n* (%)	50 (89.3)	13 (92.9)	1.0
Diabetes mellitus, *n* (%)	3 (5.4)	2 (14.3)	0.2
Dyslipidemia, *n* (%)	3 (5.4)	1 (7.1)	0.7
Related donor, *n* (%)	41 (73.2)	10 (71.4)	1.0
Nitrofurantoin, *n* (%)	27 (48.2)	7 (50.0)	0.9

Abbreviations: BMI: body mass index; CKD: chronic kidney disease.

**Table 4:** Risk factors for urinary tract infections in patients post kidney transplant.

	Urinary Infection					
	Unadjusted			Adjusted		
	Enter Method			Stepwise Method		
	HR	95% CI	*p*-Value	aHR	95% CI	*p*-Value
Female gender	8.7	2.5–29.8	<0.001	7.0	2.3–20.9	<0.001
Body mass index	1.0	0.8–1.2	0.8	--	--	--
Comorbidities	1.7	0.2–15.0	0.5	--	--	--
CKD diagnosis (yrs)	0.2	0.9–1.1	0.2	--	--	--
Nitrofurantoin	0.9	0.3–2.9	0.2	--	--	--

Abbreviations: CKD: chronic kidney disease; aHR: adjusted hazard ratio; 95% CI: 95% confidence interval. Crude HRs were obtained using the enter method. aHR was obtained using the stepwise method. The variables excluded from the final model were the body mass index, comorbidities, CKD diagnosis, and usage of nitrofurantoin.

## Data Availability

The dataset supporting the conclusions presented in this article is available on request from the corresponding author on reasonable request.
